# Materials and Techniques in Dentistry, Oral Surgery and Orthodontics

**DOI:** 10.3390/ma17133247

**Published:** 2024-07-02

**Authors:** Maria Francesca Sfondrini, Andrea Scribante

**Affiliations:** 1 Unit of Orthodontics and Pediatric Dentistry, Section of Dentistry, Department of Clinical, Surgical, Diagnostic and Pediatric Sciences, University of Pavia, 27100 Pavia, Italy; francesca.sfondrini@unipv.it; 2 Unit of Dental Hygiene, Section of Dentistry, Department of Clinical, Surgical, Diagnostic and Pediatric Sciences, University of Pavia, 27100 Pavia, Italy

Modern dentistry encompasses a broad spectrum of disciplines—restorative dentistry, endodontics, prosthodontics, periodontics periodontology, aesthetic dentistry, paediatric dentistry orthodontics, and oral hygiene. As such, dentists cooperate with maxillo-facial surgeons, dermatologists, otorhinolaryngologists, and other medical specialists in the provision of comprehensive dental care [1,2,3,4]. In addition to this, new materials and techniques are frequently introduced in daily clinical practice, and practitioners must undertake continuous study and research to keep abreast of these [5,6,7,8].

Therefore, this Special Issue aims to bring together contributions relating to the latest research trends in dental materials. The contributions presented in this volume concern the use of magnetic resonance imaging for orthodontic devices (Contribution 1), the effects of different adhesives on the release forces of orthodontic brackets (Contribution 2), demineralisation around different orthodontic attachments (Contribution 3), and the torque transmission of different wire bracket systems (Contribution 4). Furthermore, the predictability of treatments with transparent aligners (Contribution 5) and the shape memory properties of their polymers were analysed (Contribution 6). Additionally, the application of fluoride and vitamin D on deciduous teeth (Contribution 7), the influence of various enamel-cleaning systems (Contribution 8), and the use of lasers for the removal of ceramic artefacts were evaluated (Contribution 9). Lastly, the role of microscopic instrumentation in the analysis of dental material structure was also reviewed (Contribution 10).

Future studies that continue to promote research towards new interesting goals are certainly desirable. New lines of research may be explored in the future by studying biomimetic materials for restorative dentistry and prosthetics [9,10,11,12], remineralising agents [13,14,15,16], and adjuvant techniques based on laser or ozone [17,18,19,20,21]. Future reports on new technologies aimed at reducing pain and improving the doctor–patient relationship should also be considered [22,23]. Additionally, regenerative medicine will play an important role in dentistry both for hard and soft tissues [24,25,26].

Another major chapter in the future of dental materials research is likely to be 3D printing technologies. Recently introduced features allow for a fully digital workflow, which allows for more precise therapies and a reduction in treatment time [27,28,29].

Finally, artificial intelligence-based technologies are widely developing in the dental field; therefore, these features should be explored and studied in depth in order to understand their important role in clinical management. Diagnosis, therapy, and material selection could benefit from the evaluation and informed use of this technology [30,31,32,33].

Indeed, analysis of the chemical, physical, and mechanical characteristics of dental materials used in general dentistry, oral surgery, and orthodontics, together with basic and translational research studies, mechanical analyses, clinical trials, and literature reviews, will lead to the development of new working methods that aid the clinician and are more favourable for the patient.
